# Cardiac Tamponade Unmasking Recurrent Ovarian Cancer

**DOI:** 10.7759/cureus.15464

**Published:** 2021-06-05

**Authors:** Andrii Maryniak, Filip Oleszak, Patrick Biskupski, Perry Wengrofsky, Yi-Chun Lee

**Affiliations:** 1 Internal Medicine, State University of New York (SUNY) Downstate Medical Center, Brooklyn, USA; 2 Internal Medicine, New York City (NYC) Health + Hospitals/Lincoln Medical Center, Bronx, USA; 3 Cardiology, Rutgers Health/New Jersey Medical School, Newark, USA; 4 Gynecologic Oncology, State University of New York (SUNY) Downstate Medical Center, Brooklyn, USA

**Keywords:** cardiac tamponade, pericardial effusion, ovarian cancer, pericardiostomy, malignancy

## Abstract

Pericardial disease is a common manifestation of malignancy. Gynecologic malignancies such as ovarian cancer rarely present with cardiac involvement. Cardiac tamponade may be the initial presentation of malignancy in as many as half of pericardial disease cases. We report the case of a 60-year-old female with known ovarian adenocarcinoma, who achieved initial success with tumor debulking and adjuvant chemotherapy but was lost to follow-up. She presented again three years later with new-onset dyspnea and described a syncopal episode. A chest radiograph showed an enlarged cardiac silhouette and bilateral pleural effusions. Transthoracic echocardiography revealed a large pericardial effusion with diastolic collapse of the right atrium and ventricle, consistent with tamponade physiology. Subxiphoid pericardiocentesis and pigtail drain were placed under fluoroscopy with resolution of symptoms and no recurrence. Neoplastic etiology was confirmed by immunocytochemistry on cell block positive for PAX-8. As an adjunct or alternative to cytologic evaluation, diffusion-weighted magnetic resonance imaging and calculation of the apparent diffusion coefficient can be used to differentiate between malignant and benign effusions. Malignant pericardial effusion in ovarian cancer is a treatable oncologic emergency where timely diagnosis and management may facilitate palliation and prolong life.

## Introduction

In the United States, ovarian cancer is the second most common gynecologic malignancy and the most common cause of gynecologic cancer death. It is responsible for approximately 14,000 deaths each year [1,2]. Notably, 95% of ovarian malignancies are epithelial in origin and usually occur in women of older age, with a median age of 63 years at diagnosis [3]. Other risk factors include a history of early menarche or late menopause, and genetic predisposition [4]. While malignant involvement of the pericardium is detected in 1% to 20% of cancer cases in autopsy studies, gynecologic malignancies rarely present with metastases to the heart [5]. The most common metastatic tumor involving the pericardium is lung cancer. Others include breast and esophageal cancer, melanoma, lymphoma, and leukemia [5,6]. We report a case of ovarian adenocarcinoma with cardiac metastasis, resulting in a symptomatic pericardial effusion with tamponade physiology, as the initial clinical manifestation of recurrent malignancy.

## Case presentation

History

A 60-year-old G3P3003 African American female with a diagnosis of ovarian cancer, underwent total abdominal hysterectomy with bilateral salpingo-oophorectomy (TAH-BSO) and tumor debulking in 2011, adjuvant carboplatin-paclitaxel chemotherapy between 2011 to 2013, and re-treatment with platinum in 2016 for recurrence. After initial success, she was lost to follow up in 2017. Three years later, she presented to the emergency department complaining of new-onset exertional dyspnea and fatigue lasting five days. She also reported a syncopal episode without prodromal symptoms a day prior to presentation. She lost consciousness for approximately 15 minutes with a rapid return to baseline upon awakening.

Examination

The patient appeared comfortable, in no acute distress. There was no evidence of head trauma or tongue laceration. Jugular venous distension was not appreciated. Cardiac auscultation revealed distant heart sounds and tachycardia, with a heart rate of 111 beats per minute. Blood pressure was 98/70 mmHg. Peripheral pulses were intact and symmetric. Bronchovesicular breath sounds were auscultated bilaterally. The abdomen was distended, diffusely tender to superficial and deep palpation, and a midline mass was palpated. Bowel sounds were normoactive.

Diagnostic studies

**Figure 1:**
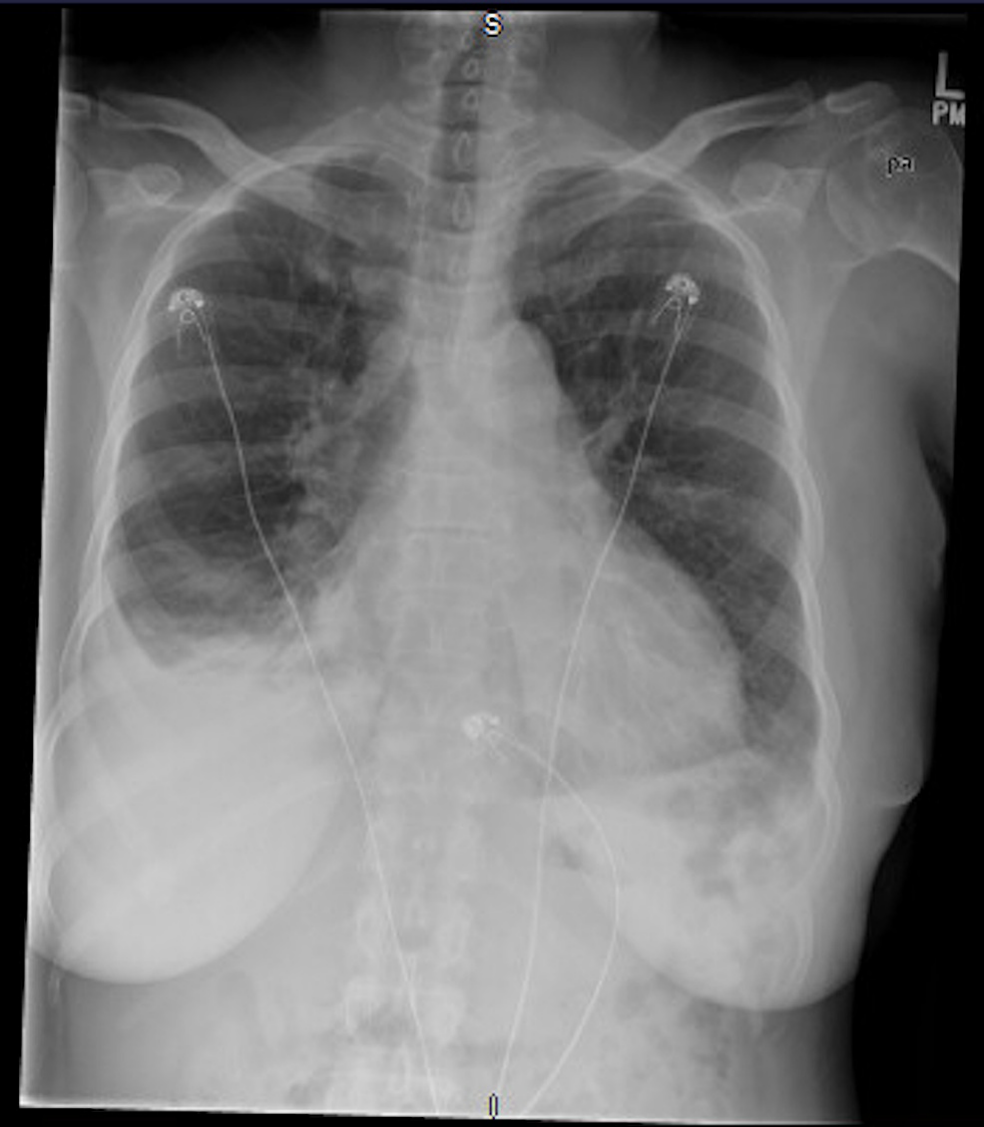
Chest X-ray showing water bottle sign.

**Figure 2:**
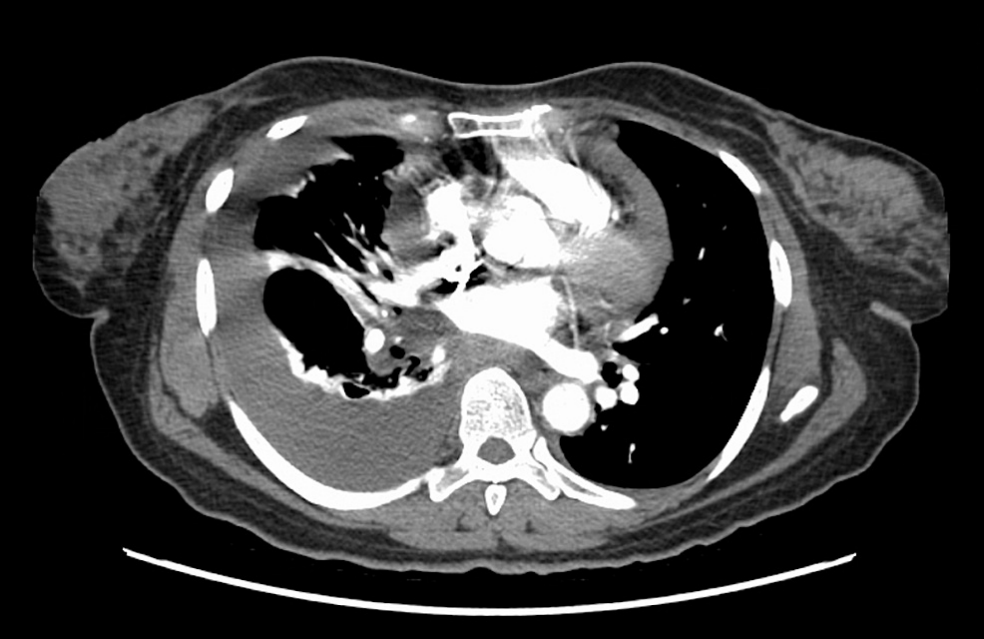
CT angiography of the chest showing pleural and pericardial effusions.

**Figure 3:**
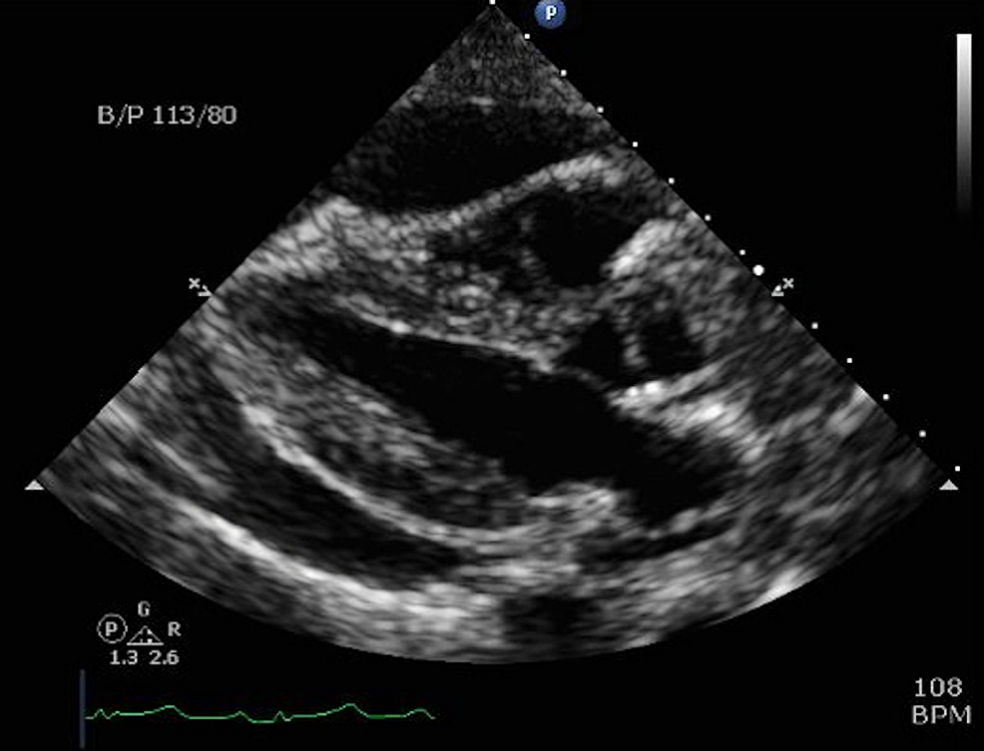
2D transthoracic echocardiography showing a large circumferential pericardial effusion in the parasternal long-axis view.

Electrocardiography showed sinus tachycardia, without ischemic changes. Serial measurements of troponin-T level were negative. Chest radiography identified bilateral pleural effusions, notably larger on the right (Figure 1). Computed tomography (CT) angiography of the chest and CT of the abdomen and pelvis revealed a large pericardial effusion (Figure 2), abdominal ascites, peritoneal carcinomatosis with bladder wall and sigmoid colon involvement, a retroperitoneal mass with encasement of the aorta, and a right hepatic lobe mass likely representing metastatic disease. Pulmonary embolism was not seen. Transthoracic echocardiography (TTE) revealed a large pericardial effusion with right ventricular and right atrial diastolic collapse (Figure 3). ProB-type natriuretic peptide (Pro-BNP) level was 137 pg/mL; however, pseudonormalization in the setting of cardiac tamponade has been documented [7]. Cancer antigen 125 (CA-125) level was 141 U/mL, representing a positive result. The patient was referred to cardiothoracic surgery for pericardiocentesis.

Procedure description

Thoracentesis yielded 900 mL of clear yellow fluid leading to interval improvement in dyspnea. Cytology was consistent with an exudative source. Pericardiocentesis via subxiphoid approach was performed under fluoroscopy. 660 mL of bloody fluid was removed from the pericardial cavity with a pericardial drain left in place to gravity. The drain was clamped and removed after there was no re-accumulation for 48 hours. Aspirated fluid was positive for adenosine deaminase (ADA) 10.7 U/L, lactate dehydrogenase (LDH) 657 IU/L, protein 5.3 g/dL, white blood cells (WBC) count 3816 cells/uL (54% lymphocytes), red blood cell (RBC) 178,125 cells/uL. Acid-fast stain was negative and bacterial cultures showed no growth. Cytology was consistent with metastatic adenocarcinoma of gynecologic/Mullerian origin, confirmed by immunohistochemistry on cell block positive for PAX-8. The patient was started on a cytostatic regimen of carboplatin, doxorubicin, and bevacizumab.

## Discussion

Pericardial effusion and cardiac tamponade lie on the same continuum of pericardial disease. In acute cardiac tamponade, pericardial fluid accumulates rapidly leading to abruptly elevated pericardial pressure which compromises diastolic ventricular filling. Retrograde accumulation of circulating volume then results in elevated jugular venous pressure, dyspnea secondary to pulmonary congestion, and decreased cardiac output leading to hypotension. Subacute cardiac tamponade is a more indolent process where pericardial fluid accumulation is slow, so patients may be asymptomatic at presentation or complain of milder symptoms such as dyspnea, fatigue, or chest discomfort [5].

Pericardial disease is a common manifestation of malignancy and may present as pericarditis (with or without constrictive physiology), pericardial effusion, or cardiac tamponade. This may occur late in the disease course but may also be the first manifestation of malignancy. In patients with malignancy, pericardial disease may be expected due to metastatic spread of the tumor, as a side effect of chemotherapy or radiation therapy, or as a result of an infectious or autoimmune process [5]. When pericardial disease presents in the absence of a known history of cancer, occult malignancy should be investigated. Imazio et al. reported on 450 cases of acute pericardial disease, and found that 33 cases (7.3%) were attributable to malignancy [8]. Among this group, besides a known history of current or prior malignancy, cardiac tamponade at presentation, lack of response to nonsteroidal anti-inflammatory drugs, and recurrent or incessant pericarditis, were identified as the primary risk factors associated with a neoplastic etiology [8]. In a separate review of 173 cases of symptomatic pericardial effusions by Ben-Horin et al., malignancy was found to be the most common cause, accounting for 58 patients (33%) [9]. Of these 58 patients, 45 had known malignant disease. In a subset of 74 patients from this cohort, where there was no identifiable cause based on history, physical exam or simple laboratory tests, new malignancy was responsible for 18% of pericardial effusions [9]. Other retrospective studies have suggested that as much as 50% of patients with malignant pericardial effusions may also have cardiac tamponade, even as the first manifestation of cancer [10].

The most common primary tumor involving the pericardium is lung cancer. Other common tumors known to metastasize to the heart include breast cancer, esophageal cancer, melanoma and hematologic malignancies [5]. Malignant involvement of the pericardium secondary to metastatic spread of gynecologic cancers, specifically ovarian cancer, is rare. This is because the primary modes of spread are intraperitoneal surface dissemination and retroperitoneal lymphatic spread [9]. Cardiac involvement is typically the manifestation of an advanced stage of ovarian cancer, albeit that it may result in the first clinical signs or symptoms of undiagnosed malignancy. 

Identification of pericardial fluid relies on TTE, which enables quick evaluation of the pericardial space and allows for semiquantitative assessment of the effusion size and its hemodynamic effects. CT and cardiac magnetic resonance imaging (MRI) can also be helpful, especially in cases of loculated effusion, pericardial thickening, or in the presence of a mass and other chest abnormalities [3,11]. After localization of fluid, pericardiocentesis or pericardiostomy are performed to provide therapeutic relief. In many cases pericardiocentesis alone is sufficient. Relapsing refractory effusions may require additional interventions such as pericardial sclerotherapy, pleuropericardial or pleuroperitoneal window, systemic chemotherapy, radiotherapy, and/or intrapericardial instillation of cytostatic agents [11]. Pleuro-pericardial window is particularly effective if the pericardial space is posteriorly located and/or segmented [12]. Injection of intrapericardial chemotherapy addresses the neoplastic etiology of malignant effusions. Cisplatin is a frequently used agent because of its efficacy against the most frequent malignancies known to cause secondary pericardial effusions, its low incidence of adverse effects when instilled locally, and its sclerosing effects [11].

Cytologic evaluation of aspirated pericardial fluid and pericardial biopsy are the main diagnostic modalities used to determine etiology. Dragoescu and Liu conducted a retrospective analysis of 128 pericardial fluid specimens collected from 113 patients over six years and determined that pericardial fluid cytology was superior in detecting malignancy compared to pericardial biopsy (71% sensitivity, 100% specificity vs. 64% sensitivity, 85% specificity. Although cytology is superior to biopsy in diagnosing metastatic carcinoma, certain tumors may go undetected in pericardial fluid [13].

As an adjunct or alternative to cytologic evaluation, the use of diffusion-weighted MRI and calculation of the apparent diffusion coefficient (ADC) has been studied to differentiate between malignant and benign pericardial effusions. Razek and Samir conducted a retrospective analysis of diffusion-weighted MRI of 41 patients with pericardial effusions and correlated their calculations of ADC with cytologic analyses [14]. They found that the calculated ADC was significantly higher in benign pericardial effusions compared to malignant effusions and that a cut-off value of the ADC can be determined to differentiate between effusion types. Overall the ADC value serves as a non-invasive imaging parameter that can be used in addition to, or in favor of, cytologic analysis to rule-in or rule-out malignancy [14]. The usefulness of measuring serum CA-125 levels for monitoring the clinical course of pericardial effusion has also been studied. Seo et al. retrospectively investigated the relationship between serum CA-125 and the presence and severity of pericardial effusions [15]. CA-125 levels were higher in patients with larger effusions and the serum level decreased when the pericardial effusion was reduced with intervention.

Perri et al. studied the long-term outcomes of patients with ovarian cancer after developing pericardial effusions [16]. They identified several cases in the literature reporting poor survival. However, the investigators also reported that patients with mild systemic manifestations of malignancy may have favorable outcomes such as prolonged survival if pericardial effusions are identified and resolved promptly. They concluded that malignant pericardial effusion in ovarian cancer is a treatable oncologic emergency where timely diagnosis and management may facilitate palliation and prolong life [16]. Overall, because neoplastic involvement of the pericardium occurs at advanced stages, morbidity and mortality are very high in both patients with known malignancies and in those patients where cancer is newly diagnosed [10].

## Conclusions

Pericardial effusions are a common sequelae of malignancy. They may occur in the setting of known history of cancer but may also be the initial clinical manifestation. When fluid in the pericardial space accumulates rapidly and overcomes the compensatory capacity of the heart to adapt to varying intracardiac pressures, this compromises diastolic filling, leading to tamponade physiology. Ovarian cancer rarely presents with pericardial involvement, only at advanced stages of disease. Echocardiography is the primary modality used to diagnose pericardial effusions. In most cases, pericardiocentesis is both diagnostic and therapeutic. Definitive diagnosis is obtained via cytologic evaluation. Diffusion-weighted MRI and calculation of ADC provide a non-invasive tool to differentiate between benign and malignant effusions. Measurement of serum CA-125 levels is useful to monitor the clinical course of the disease and to stratify the severity of effusion. A similar prognosis has been found for patients with known malignancy and those with newly diagnosed malignancy.
